# Subjective memory complaint and its relationship with cognitive changes and physical vulnerability of community-dwelling older adults

**DOI:** 10.1590/1980-57642018dn13-030012

**Published:** 2019

**Authors:** Daniela Dalpubel, Paulo Giusti Rossi, Mariana Luciano de Almeida, Estela Barbosa Ribeiro, Renata Araújo, Larissa Pires de Andrade, Francisco de Assis Carvalho do Vale

**Affiliations:** 1 Federal University of São Carlos Department of Nursing SP Brazil MSc. Department of Nursing, Federal University of São Carlos, SP, Brazil.; 2 Federal University of São Carlos Department of Physical Therapy SP Brazil MSc. Department of Physical Therapy, Federal University of São Carlos, SP, Brazil.; 3 Federal University of São Carlos Department of Physical Therapy SP Brazil PhD. Department of Physical Therapy, Federal University of São Carlos, SP, Brazil.; 4 Federal University of São Carlos Department of Medicine SP Brazil PhD. Department of Medicine, Federal University of São Carlos, SP, Brazil.

**Keywords:** memory complaint, cognitive impairment, physical vulnerability, elderly, informant, queixa de memória, alteração cognitiva, vulnerabilidade física, idosos, informante

## Abstract

Memory complaint (MC) is common in older adults and can be confirmed by people close to them, such as family members and caregivers. Studies show an association between MC and cognitive impairment and, hence, physical vulnerability may exacerbate MC. However, the relationship between MC and physical vulnerability is not yet clear in the literature.\

**Objective:**

to investigate the association between MC, cognitive impairment, and physical vulnerability.

**Methods:**

this is a cross-sectional study. We evaluated 100 older adults with a mean age of 65 years or over. The Memory Complaint Scale (MCS), Addenbrooke's Cognitive Examination-Revised (ACE-R), Mini-Mental State Examination (MMSE), Vulnerable Elderly Research-13 (VES-13), Geriatric Depression Scale and a sociodemographic questionnaire were applied.

**Results:**

participants were divided into two groups according to results on the MCS-A (elderly) and MCS-B (informant). Correlations were found between the MCS-A and the MMSE (p=.045/ρ=.201), ACE-R/Visual-Spatial (p=.048/ρ=.199), and ACE-R/Attention-Orientation (p=.026/ρ=.223). For the MCS-B, correlations were found with total score on the ACE-R (p=.044/ρ=-.202) and the ACE-R/Visual-Spatial (p=0.003/ρ=-.291).

**Conclusion:**

MC reported by the informant indicate the need to assess, in more depth, the cognition of the older adult. Thus, for clinical practice, screening of MC through an informant is advised.

## RESUMO

A queixa de memória (QM) é comum em idosos e pode ser confirmada por pessoas próximas a ele, como familiares e cuidadores. Estudos apontam associação entre QM e alterações cognitivas e, nesse sentido, a vulnerabilidade física poderia exacerbá-la. Porém, a relação entre QM e vulnerabilidade física ainda não está clara na literatura.

**Objetivo:**

investigar a relação entre QM, alterações cognitivas e vulnerabilidade física.

**Métodos:**

trata-se de um estudo transversal. Foram avaliados 100 idosos com idade igual ou superior a 65 anos. Utilizou-se a Escala de Queixa de Memória (EQM), Exame Cognitivo de Addenbrooke - Revisado (ACE-R), Mini Exame do Estado Mental (MEEM), Vulnerable Elders Survey-13 (VES-13), Escala de Depressão Geriátrica e questionário sociodemográfico.

**Resultados:**

os participantes foram divididos em dois grupos de acordo com os resultados da EQM formas A (idoso) e B (informante). Encontrou-se correlação entre a EQM-A e MEEM (p=.045/ρ=.201), ACE-R Atenção e Orientação (p=.026/ρ=.223) e ACE-R/Visual-Espacial (p=.048/ρ=.199). Na EQM-B encontrou-se correlação entre pontuação total do ACE-R (p=.044/ρ=-.202) e ACE-R/Visual-Espacial. (p=.003/ρ=-.291).

**Conclusão:**

o relato de QM a partir do informante aponta a necessidade de avaliação mais aprofundada da cognição dos idosos. Assim, para a prática clínica, o rastreio de QM do informante é aconselhado.

Aging may be accompanied by a decline in cognitive functions, with memory difficulty often reported by the elderly.^1^ The term memory complaint (MC), usually called a subjective memory complaint, is generally used to refer to a report of self-perceived memory problems, which may or may not be confirmed by people close to the elderly, known as informants.^2^^,^^3^

MC is associated with anguish, and reduced mental health, well-being, and quality of life.^4^ Regarding cognition in the elderly, MC is associated with an increased risk of future cognitive decline and constitutes one of the diagnostic criteria for different types of dementia.^5^^,^^6^MC has been identified as one of the main indicators for the evaluation, identification, and classification of mild cognitive impairment.^7^ In addition, people with MC have 2.5 to 6.5 times the risk of developing dementia when compared to individuals without MC.^2^^,^^8^^,^^9^

The prevalence of MC increases with advancing age and research investigating the definition of these complaints is necessary to clarify their clinical significance and relation to cognitive decline, since decline in some cognitive functions during aging is frequent, even in the absence of neurological disease.^1^ Cognitive alterations can be measured by means of neuropsychological tests that evaluate several areas of cognition in order to aid the neurological diagnosis.^10^^,^^11^ Decreased cognitive ability in the elderly is associated with the decline in specific skills, such as abstract thinking, speed of thought processing, memory aspects, and executive function.^12^ In addition, cognitive impairment is more frequent in physically vulnerable elderly individuals.^13^

Physical vulnerability also includes an important and multidimensional aspect to be evaluated in the aging process; understood as a process of risk in general health condition, resulting from social, economic, family, psychological, cognitive, and/or physical resources.^14^^,^^15^ Due to the action of these genetic-biological, psychological, and sociocultural variables, older adults represent a group especially exposed to vulnerability, which differs from other phases of the life cycle.^13^^-^15 It is not yet clear how physical vulnerability may relate to MC since the available evidence is only associated with objective cognitive impairment. In view of these aspects related to cognitive impairment and physical vulnerability, which are conditions present in aging that can manifest in different dimensions, the objective of the present study was to investigate the relationship between MC and objective cognitive alterations and physical vulnerability.

## METHODS

### Study design and participants

This is a cross-sectional study that evaluated older adults (≥65 years) living in the community, divided into two groups (with memory complaint and without memory complaint) according to the results on the Memory Complaint Scale (MCS), forms A and B.^16^ The MCS consists of seven questions and aims to systematically screen MC through the two-part application: MCS-A, applied directly to the evaluated participant; and MCS-B, applied to his/her companion/informant reporting on the participant's memory.

Participants were recruited from a random stratified proportional sample of individuals living in the municipality of São Carlos, São Paulo state, Brazil. The strata and their quantity were defined by the number of combinations of gender categories and age groups, from the pre-defined age, taking as a reference the subdivision used by the Brazilian Institute of Geography and Statistics and the information available on the data collected in the 2010 Census.^17^

Inclusion criteria were: elderly people of any gender aged 65 years or over, an informant who knew the participant sufficiently to answer the questionnaires and confirm information related to the elderly individual. Participants with mental disorders; uncorrected auditory or visual deficits that made the tests impossible to complete; and a score ≥5 on the Geriatric Depression Scale (GDS)^18^ were excluded. The study was approved by the ethics and research committee of the institution and all participants signed the free and informed consent form (CAAE: 02878312.0.0000.5504 and approval 92,000 / 2012). All ethical aspects provided for by Resolution 466/12 MS and regulated by the National Health Council were appropriately observed and respected.

### Data collection instruments

Data collection was performed from January 2015 to May 2016 by three suitably trained interviewers. The interviews were carried out in the households of the elderly, individually and in an environment with low visual and auditory stimuli. All tests were explained in a simple, objective, and clear way to the participants. All the elderly participants were evaluated by a neurologist who is a specialist in the area of cognitive disorders and dementias. Anamnesis, memory complaint evaluation, cognitive evaluation, and physical vulnerability assessments were performed.

Descriptive variables were evaluated using a questionnaire collecting data on age, sex, marital status, years of study, and presence of depressive symptoms (score >5 on the Geriatric Depression Scale - GDS-15).^18^ In order to classify the sample into economic classes, the *Critério de Classificação Econômica Brasil* (CCEB)^19^ was used.

### Cognition and physical vulnerability

For the cognitive evaluation, the Mini-Mental State Examination (MMSE)^20^ and the Addenbrooke's Cognitive Examination-Revised (ACE-R), validated for the Brazilian population,^21^ were used. The ACE-R is an instrument with high sensitivity and specificity for detecting mild stage dementia. The instrument contains five domains, each with a specific score, where final score ranges from 0 to 100 points.^21^^,^^22^

Physical vulnerability was assessed by the Vulnerable Elders Survey (VES-13), using a validated version for the Brazilian population.^13^ This instrument specifically seeks to identify physically vulnerable elderly individuals and its components include individual aspects relevant to the health of older adults, such as age, health self-assessment, mobility, and functional capacity.^13^

### Statistical analysis

A level of significance of α=0.05 was adopted and the software used for statistical analysis was SPSS 20.0 (IBM, Chicago, Illinois). The sample calculation was performed using G * Power software 3.1.9.2, based on the correlation test and adopting an effect size of 0.6, significance level of 5%, and power of 80%. The results demonstrated the requirement for 94 volunteers in the total sample of the study.

The normality of the data was verified using the Kolmogorov-Smirnov test. Intergroup analyses were performed using the Mann-Whitney test and, the Spearman correlation test was used to determine the correlation between the variables. Intragroup analyses were performed using the Wilcoxon test. The general rule was used to interpret the size of a correlation coefficient of Mukaka^23^ for the classification of correlations.

## RESULTS

In total, 117 elderly people were evaluated, of which five were excluded because they had a diagnosis of dementia and 12 due to depressive symptoms according to the GDS (≥5 points). Thus, for the analysis, 100 elderly were included in the study. The participants of the present study were predominantly female (68%), had a mean age of 74.3 (±7.2) years, and low educational level, as shown in Table 1. The majority of the older adults practiced the catholic religion (82%), were married/stable union (65%), and belonged to social classes B2 and C1(33% and 29% of sample, respectively).

**Table 1:** Sociodemographic characteristics of final sample of people aged 65+ living in a rural city in São Paulo state, Brazil, 2016.

Variables n=100	Mean	Min	Max	Std Dev	Std Error
Age (years)	74.32	65.00	95.00	7.24	0.72
Height (m)	1.59	1.40	1.80	0.08	0.00
Weight (kg)	68.41	40.00	118.00	13.79	1.37
BMI (kg/cm^2^)	27.13	17.77	41.09	5.41	0.54
Education (years)	5.12	0.00	19.00	4.21	0.42
Medication (n)	1.90	1.00	3.00	0.33	0.03

BMI: Body Mass Index.

Source: Researcher's database.

The participants were divided into two groups according to the results on the MCS form, parts A and B. Thus, one group comprised participants with memory complaint (MC Group) and the other group consisted of individuals without memory complaint (NMC Group).

According to the intergroup analyses, performed using the Mann-Whitney test, of the results on the MCS-A (applied to elderly), no statistically significant differences between the groups were found (Table 2). Therefore, this result indicates that, although elderly reported memory complaints, these findings did not correlate with the absolute values on the cognitive tests.

**Table 2:** Clinical characteristics of final sample of people aged 65+ living in a rural city in São Paulo state, Brazil, according to the MCS-A. 2016.

Variables	MCS-A		
	MC n=45	NMC n=55	P value
Age (years)	73.8 (±7.2)	74.7 (±7.3)	.419
Education (years)	5.4 (±3.6)	4.8 (±4.6)	.156
Female	34 (75.6%)	34 (61.8%)	.145
VES-13	1.7 (±2.3)	2 (±2.6)	.983
ACE-R - MMSE	24.8 (±3.4)	23.4 (±2.6)	.192
ACE-R (total)	70.4 (±13.6)	64.4 (±18.4)	.143
ACE-R Attention and Orientation	15.4 (±2.3)	14.6 (±2.7)	.123
ACE-R Fluency	6.9 (±2.4)	6.5 (±3.4)	.329
ACE-R Memory	15.5 (±4.5)	14.7 (±5.1)	.472
ACE-R Language	21.0 (±4.3)	18.7 (±6.4)	.157
ACE-R Visual-Spatial	11.4 (±3.3)	9.7 (±4.2)	.051

MC: older adults with memory complaint; NMC: older adults without memory complaint; MCS-A: Memory Complaint Scale form A; VES-13: Vulnerable Elders Survey; MMSE: Mini-Mental State Examination; ACE-R: Addenbrooke's Cognitive Examination-Revised. Data expressed as mean (standard deviation)

* p<0.05.

Source: Researcher's database.

The MC and NMC groups were evaluated by the MCS-B (applied to family member/caregiver) and the Mann-Whitney test was also used. There was homogeneity of the sociodemographic data, however, statistically significant differences were observed for total ACE-R score (p=0.020) and Visual-Spatial domain (p=0.001), as shown in Table 3. The elderly had statistically significantly lower scores on the total cognitive evaluation of the ACE-R and visual-spatial domain.

**Table 3:** Clinical characteristics of final sample of people aged 65+ living in a rural city in São Paulo state, Brazil, according to the MCS-B. 2016.

Variables	MCS-B		
	MC n=36	NMC n=64	P value
Age (years)	75.1 (±6.9)	73.8 (±7.4)	0.290
Education (years)	4.8 (±4.9)	5.2 (±3.7)	0.252
Female	25 (69.4%)	43 (67.2%)	0.817
VES-13	2.0 (±1)	1.8 (±2.4)	0.698
MMSE	23.1 (±5.1)	24.5 (±3.5)	0.235
ACE-R (total)	62.1 (±16.5)	70 (±16.0)	0.020*
ACE-R Attention and Orientation	14.5 (±3.1)	15.2 (±2.2)	0.509
ACE-R Fluency	6.1 (±2.9)	7.8 (±2.9)	0.135
ACE-R Memory	14 (±4.2)	15.6 (±5.1)	0.122
ACE-R Language	28.4 (±6.2)	20.5 (±5.2)	0.055
ACE-R Visual-Spatial	8.9 (±3.5)	11.4 (±3.9)	0.001*

MC: older adults with memory complaint; NMC: older adults without memory complaint; MCS-B: Memory Complaint Scale form B; VES-13: Vulnerable Elders Survey; MMSE: Mini-Mental State Examination; ACE-R: Addenbrooke's Cognitive Examination-Revised. Data expressed as mean (standard deviation).

* p<0.05.

Source: Researcher's database.

The correlation test verified possible correlations between the MCS-A, MCS-B, and other variables. Statistically significant associations were identified between the MCS-A and ACE-R Attention and Orientation domain (p=0.026 / ρ=.223); the MCS-A and Visual-Spatial domain of the ACE-R (p=0.048 / ρ=.199); and the MCS-A and MMSE (p=0.045 / ρ=.201).

Correlations were also identified between the MCS-B and total ACE-R score (p=0.044 / ρ= -. 202); and between the MCS-B and Visual-Spatial domain of the ACE-R (p=0.003 / ρ= -291). There was also a correlation between the VES-13 and total ACE-R score (p=0.001 / ρ= -. 336); the VES-13 and ACE-R Memory domain (p=0.002 / ρ= -313); the VES-13 and ACE-R Fluency domain (p=0.001 / ρ= -325); the VES-13 and ACE-R Language domain (p=0.001 / ρ= -328); and the VES-13 and MMSE (p=0.006 / ρ= -271), as depicted in Table 4.

**Table 4:** Results of correlations with Spearman's test. Brazil, 2016.

Variables	MCS-A	MCS-B	VES-13
VES-13	.350	.335	1
MMSE	.045* ρ=.201	.168	.006* ρ= -.271
ACE-R (total)	.130	.044* ρ= -.202	.001* ρ=-.336
ACE-R Attention and Orientation	.026* ρ=.223	.282	.102
ACE-R Memory	.584	.357	.002* ρ= -.313
ACE-R Fluency	.223	.394	.001* ρ= -.325
ACE-R Language	.234	.057	.001* ρ= -.328
ACE-R Visual-Spatial	.048* ρ=.199	.003* ρ= -.291	.229

Data presented with r-value. MCS: Memory Complaint Scale; ACE-R: Addenbrooke's Cognitive Examination-Revised; MMSE: Mini-Mental State Examination; VES-13: Vulnerable Elders Survey.

* p<0.05. ρ-value for rho.

Source: Researcher's database.

## DISCUSSION

The aim of the present study was to investigate the relationship between MC, cognitive impairment, and physical vulnerability in elderly residents of a Brazilian city, based on a sample from a population-based study. The results of this study revealed positive correlations between MC reported by the elderly and cognitive alteration on the screening tests used. Regarding the results of informants´ reports of MC in the elderly individuals, these showed negative correlations with scores on cognitive screening tests, i.e. when the informant reported a memory complaint in the elder, the individual actually scored lower on the cognitive tests.

The elderly in the sample were predominantly female, had a mean age of 74.3 years, and average schooling of four years. These data are consistent with national and international studies of population samples evaluating MC in community-dwelling elderly individuals.^24^^-^28

Barbosa et al.^29^ evaluated the physical, social, and programmatic vulnerability of elderly in the city of João Pessoa - Brazil, and found a significant association between physical vulnerability and MC. This result differs from the data found in the present study, in which there was no association between MC (reported by elderly or informant) and the VES-13. The findings of the study by Barbosa et al.^29^ cannot be fully compared to the present study, since these authors evaluated self-reported MC based on a single question (“Do you have a memory problem?”). It should be noted that physical vulnerability is associated with incapacitating morbidities, and functional, psychological, physical, and general well-being decline in the elderly.^30^^-^32 Also, in the present study, participants with a diagnosis of dementia were excluded.

In the analysis of the variables physical vulnerability and cognition, negative correlations were found between the VES-13 and ACE-R and between the VES-13 and MMSE whereby, the lower the score of the elderly on the screening tests, the higher their VES-13 score. Similar findings were reported in the study by Maia et al.,^13^ which assessed physical vulnerability and cognition in the elderly using the VES-13 and MMSE. The similarity of these data may be due to the similarity of the studied population with regard to age, education and population samples. It is also noteworthy that older adults are especially exposed to physical vulnerability, which generates physical, emotional, and mental harm.^14^^,^^15^

Regarding the MCS-A, the mean values for the MMSE were similar, 24.8 points for the MC group and 23.4 points for the NMC group. These data are consistent with those of Paulo and Yassuda^33^ and Silva et al.,^34^ who evaluated MC using the Memory Complaint Questionnaire (MAC-Q) instrument, cognitive performance using the MMSE, and depressive symptoms using the GDS. The similarity between our findings and the aforementioned studies may be due to the characteristics of the populations: mostly female individuals, aged between 65 and 74 years, with mean schooling of four years, and mean score on the MMSE of 23 points.^33^^,^^34^

Regarding ACE-R, the means were higher in the group with MC compared to the group without MC, scoring 70.4 and 64.4 points, respectively, where older adults who had more complaints also had higher scores. These results differ from those in the study of Pendlebury et al.^35^ analyzing the relationship between MC and cognition. The group used cognitive screening instruments the Montreal Cognitive Assessment (MoCA), ACE-R, and MMSE in elderly with mild cognitive impairment (MCI) and cerebrovascular disease. In the MCI group, the participants had a mean ACE-R score of 89 points.^35^ This difference can be justified by the influence of education in the elderly of the present study, which was lower than that of Pendlebury et al.,^35^ given educational level influences total score on the ACE-R.^21^ In addition, the cited study did not apply a specific scale for MC, only a single question (“Do you think you have more problems with your memory than most people?”).

A positive correlation was found between the MCS-A and the ACE-R Attention and Orientation domain (p=.026; ρ=.223) and Visual-Spatial domain (p=.048; ρ=.199), and between the MCS-A and the MMSE (p=.045; ρ=.201). These data are in line with the findings of two other studies in which MC and cognitive alterations were evaluated.^34^^,^^36^ The first study evaluated the relationship between MC, depressive symptoms and cognitive performance, using the MAC-Q in 301 elderly people living in São Paulo. The second study evaluated MC and its relationship with the MMSE in 152 elderly people, asking the following question: “Have you had memory difficulties that hinder your routine?”. The similarity with our results may stem from the self-awareness of the elderly without dementia about changes in their cognitive functions, even when specific tests are not yet able to detect possible cognitive decline.^37^

For scores on the MCS-B, the mean value for the MMSE was 23.1 points for the MC group and 24.5 points for the NMC group. These scores were lower than those found by Abreu,^38^ who used the Informant Questionnaire of Cognitive Decline in the Elderly (IQCODE) to measure MC of the elderly relative of the informant, and found an average of 29 points on the MMSE. The mean values for the ACE-R were 62.1 points in the MC group and 70 points in the NMC group.^38^

A statistically significant difference was found between the MCS-B and total ACE-R score and between the MCS-B and the Visual-Spatial domain of the ACE-R. These results corroborate the findings of Hancock and Larner,^39^ who assessed the MC of the elderly through an informant using the IQCODE, the study of Gifford et al.,^40^ who verified whether the MC reported by the informant was related to the overall cognitive decline of the elderly, and Hsu et al.^41^ who investigated whether the informant's score using The Prospective and Retrospective Memory Questionnaire (PRMQ) instrument was associated with cognitive measures of the elderly. Both Gifford et al.^40^ and Hsu et al.^41^ found significant associations of the decline in the objective measures of short-term memory and general cognitive capacity of the elderly with the MC reported by the informant.

The most notable finding of the present study was the informant's report on the MC of the older adult. When the informant reported a major complaint regarding the participant's memory, the participant actually had lower scores on the cognitive tests. These data suggest, therefore, that the MC reported by the informant may be useful in clinical practice to confirm a possible cognitive disorder, since often, an older adult with an impairment may not perceive the decline.^42^

Some limitations of this study should be noted. The sample cannot be generalized since it is region-specific. Stress and anxiety were not assessed in the elderly or informants, and these data may influence the subjective responses on the questionnaires. However, the overall cognitive assessment and neurologist assessment minimize this factor. Another relevant element is that, although the MCS is a new scale, it was easy and quick to apply. This was the first Brazilian study to associate memory complaints evaluated by a self-report scale with the physical vulnerability of elderly from the community. Longitudinal studies exploring these issues are suggested for future research.

In conclusion, aging is a singular, universal, and heterogeneous process of change. Therefore, it is necessary that attention be focused on all dimensions of the individual and all information related to the health of the elderly be considered relevant. Regarding the physical vulnerability of the elderly, there is a clear need to use appropriate scales, such as the one used in the present study. For clinical practice, it is advisable to screen the elderly, since this group is especially exposed to physical vulnerability which poses a health risk. The MC reported by the companion is an important aspect to guide the evaluation of the elderly, even in the absence of a complaint by the older adult subject. Therefore, when the informant indicates evidence of change in the memory of an older adult, a thorough cognitive assessment is recommended.
